# Pan‐gammaherpesvirus control via a broadly neutralising gB antibody

**DOI:** 10.1002/ctm2.70805

**Published:** 2026-09-08

**Authors:** Zi‐Ying Jiang, Peng‐Lin Li, Chu Xie, Cong Sun

**Affiliations:** ^1^ State Key Laboratory of Oncology in South China Guangdong Provincial Clinical Research Centre for Cancer Guangdong Key Laboratory of Nasopharyngeal Carcinoma Diagnosis and Therapy Sun Yat‐sen University Cancer Centre Guangzhou P. R. China

1

Gammaherpesviruses are globally endemic and drive a substantial burden of malignant and autoimmune diseases. Their lifelong latency and high asymptomatic carriage rate have long obscured their full public health impact. Epstein–Barr virus (EBV) and Kaposi's sarcoma–associated herpesvirus (KSHV), the only known human oncogenic herpesviruses, infect over 90% of the global population.^1^ EBV contributes to nasopharyngeal carcinoma, gastric cancer, multiple lymphomas, and is strongly associated with multiple sclerosis.^1^ In immunocompromised populations—including solid organ transplant recipients, people living with HIV, and patients receiving chemotherapy or cellular therapies—EBV and KSHV co‐infection can trigger primary effusion lymphoma (PEL), an aggressive B‐cell malignancy with poor clinical outcomes.^2^, ^3^ Livestock pathogens such as alcelaphine herpesvirus 1 (AlHV‐1), ovine herpesvirus 2 (OvHV‐2) and murid herpesvirus 68 (MHV68) also cause significant economic losses and carry zoonotic potential.

Despite extensive mechanistic understanding of gammaherpesvirus infection, no licensed antiviral agents or preventive vaccines are currently available.^4^ Conventional vaccine strategies have focused on single‐virus surface antigens—such as EBV gp350, gH/gL or gB—but have been hampered by sequence divergence across genera and the viruses’ ability to persist in latent states.^4^ Our recent study identified Fab5, a broadly neutralising antibody targeting the conserved fusion glycoprotein gB of gammaherpesviruses.^5^ Fab5 recognises a shared vulnerability in gB domain I (DI) and confers robust protection against EBV, KSHV, MHV68, and rhesus lymphocryptovirus (rhLCV) in animal models.^5^ This advance addresses a longstanding therapeutic gap and reshapes the framework for prevention and treatment of gammaherpesvirus‐associated diseases.

All herpesviruses share a conserved structural architecture and entry machinery. The envelope glycoproteins gH/gL and gB are essential for infection: gH/gL initiates the fusion cascade, while gB executes membrane fusion.^6^ Notably, gB exhibits exceptional sequence conservation within and across herpesvirus subfamilies and genera, making it a more promising universal target than gH/gL for gammaherpesviruses. Given the lack of broadly neutralising antibodies targeting gammaherpesvirus gB, we performed a comprehensive screen of published and proprietary gB‐directed antibodies, yielding Fab5 (Figure 1). This murine antibody was originally isolated from mice immunised with an EBV chimeric nanoparticle vaccine developed by our group.^7^ Fab5 binds gB from five genera—*Lymphocryptovirus*, *Rhadinovirus*, *Macavirus*, *Manticavirus* and *Percavirus*—via interactions with invariant DI residues including Q140, R265, G266 and T267.^5^ Parallel work has described 16F9, a broadly protective alphaherpesvirus antibody that targets the gB DI of herpes simplex virus 1, herpes simplex virus 2, pseudorabies virus and varicella‐zoster virus.^8^ Together, these findings establish gB DI as a conserved functional hotspot for the development of broadly protective antibodies and vaccines.

**FIGURE 1:**
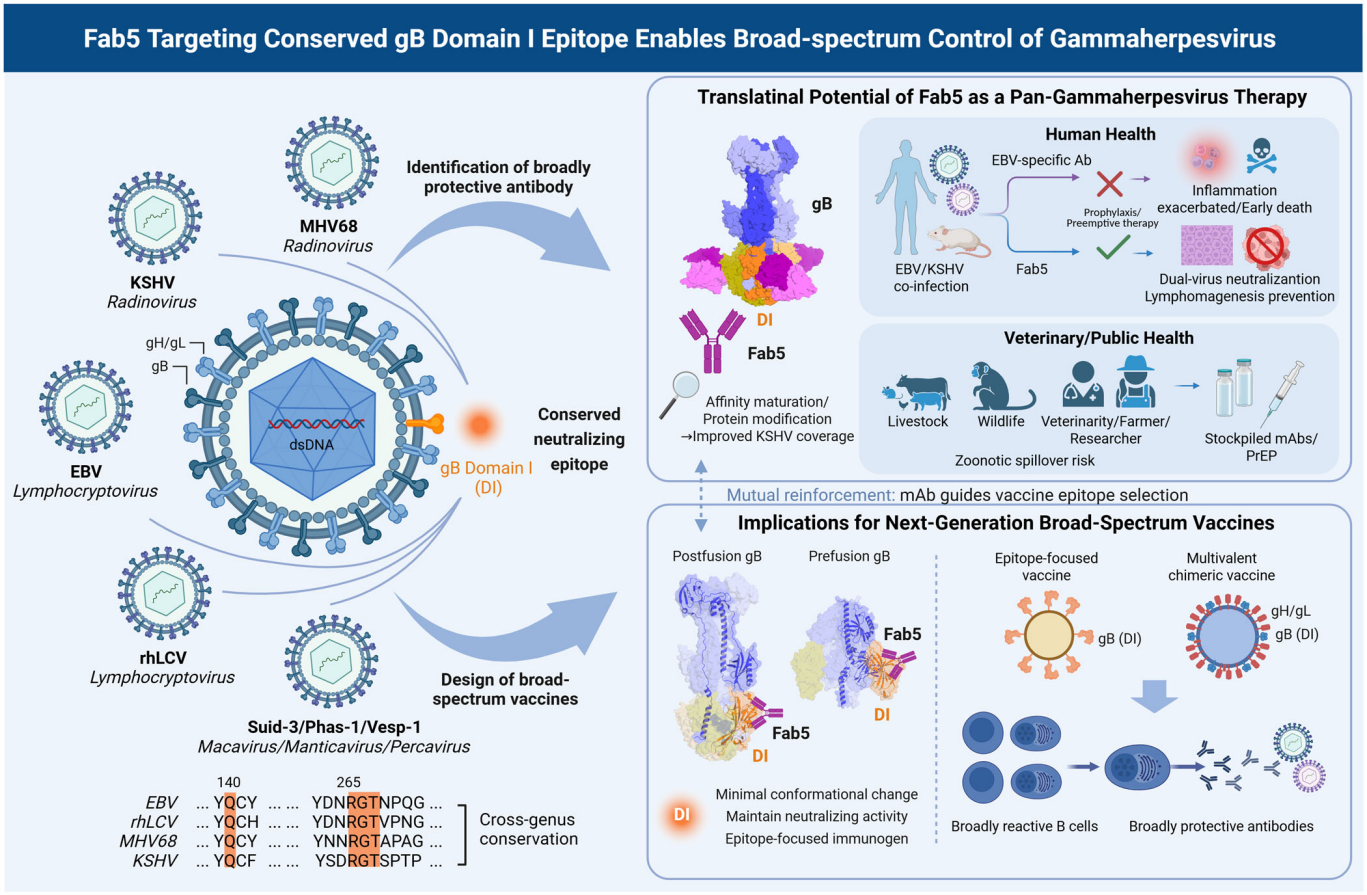
Fab5 targeting conserved gB domain I epitope enables broad‐spectrum control of gammaherpesvirus. Structural analyses reveal that the gB domain I (DI) is invariantly conserved across diverse gammaherpesvirus genera (left). The broadly neutralising antibody Fab5 targets this cryptic site, delivering dual neutralisation of Epstein–Barr virus (EBV) and Kaposi's sarcoma–associated herpesvirus (KSHV) that prevents B‐cell lymphomagenesis in co‐infected humanised mice, which rescued the lethal exacerbation observed with single‐virus‐specific antibodies (upper right). Capitalising on the minimal conformational change of DI between pre‐ and post‐fusion states, this epitope‐focused insight redefines vaccine design: stabilised DI immunogens and DI‐centred multivalent constructs offer a scalable strategy for prophylactic vaccines against both established human pathogens and emerging zoonotic threats (lower right).

In humanised mouse models of EBV/KSHV co‐infection, Fab5 demonstrated pronounced therapeutic benefits.^5^ The EBV‐specific antibody 3A3 reduced EBV viral load but failed to control KSHV; this monotherapy exacerbated systemic inflammation and accelerated mortality relative to PBS controls.^5^ In contrast, Fab5 neutralised both EBV and KSHV, reduced splenic viral loads, and prevented B‐cell lymphomagenesis.^5^ This finding underscores a critical principle: in co‐infected hosts, selective targeting of a single virus can disrupt viral equilibrium and precipitate adverse clinical outcomes. For immunosuppressed patients, EBV and KSHV reactivation remains a leading cause of lymphoproliferative disease. Standard clinical management relies predominantly on immunosuppression reduction and repurposed antiviral agents, with no therapies that directly target conserved viral entry mechanisms. Fab5 supports a multifaceted clinical strategy spanning pre‐exposure prophylaxis, preemptive intervention, and adjunctive therapy. Humanised Fab5 could be deployed as pre‐exposure prophylaxis for high‐risk cohorts, or as early intervention upon co‐infection diagnosis to prevent lymphoma onset. It may also serve as a preemptive therapeutic when rising EBV or KSHV DNAemia indicates impending disease, curbing viral expansion before symptomatic progression. This approach addresses a critical unmet need for patients receiving cellular or immunosuppressive therapies. Additionally, Fab5 provides a rationale for adjunctive treatment of virus‐associated malignancies, positioning it as a versatile platform for managing gammaherpesvirus‐driven disease across clinical contexts.

Fab5's broad protective range also supports its potential as a countermeasure against zoonotic spillover. As human activity encroaches on wildlife habitats, viruses such as MHV68—already capable of infecting human cell lines—and livestock pathogens including bovine herpesvirus 4 and AlHV‐1 represent emerging zoonotic threats. Fab5 could be stockpiled for emergency passive immunisation during outbreaks, or used as pre‐exposure prophylaxis for veterinarians, livestock workers, and wildlife researchers. Affinity maturation and protein engineering can further optimise Fab5, enhancing its potency against weaker targets such as KSHV and expanding coverage across the gammaherpesvirus family.

The high conservation of gB DI and its ability to elicit broadly neutralising antibodies make it a compelling target for next‐generation broad‐spectrum vaccine design. DI undergoes minimal conformational change between prefusion and postfusion states and remains accessible on the prefusion trimer.^5^ These findings challenge the prevailing paradigm that prefusion‐stabilised antigens are universally superior for herpesvirus vaccine development;^9^ postfusion gB, with its superior stability and expression yield, represents an equally effective immunogen. Future broadly protective vaccine strategies should prioritise epitope‐focused design centred on stabilised gB DI constructs that faithfully present the conserved neutralising epitope—an approach validated across challenging viral targets including respiratory syncytial virus F protein, influenza hemagglutinin, and coronavirus spike proteins.^10^ Building on this framework, multivalent chimeric immunogens can be engineered by incorporating complementary epitopes onto a DI‐centered scaffold to broaden and amplify protective antibody responses. Given Fab5's modest potency against KSHV, integrating additional protective epitopes beyond DI could target multiple steps in the viral entry pathway, enhancing overall neutralisation breadth and overcoming current limitations in cross‐genus coverage. This DI‐centric, structure‐guided roadmap supports the development of next‐generation broad‐spectrum vaccines against both established human pathogens and emerging zoonotic gammaherpesviruses, and may be extensible to other herpesvirus subfamilies.

Fab5 exemplifies how structural virology and antibody engineering can address longstanding challenges in herpesvirus intervention. By identifying a conserved, cross‐genus vulnerability, it enables broad prophylaxis, preemptive therapy, and next‐generation vaccine development. Equally importantly, it establishes co‐infection biology as a core consideration in therapeutic design. As gammaherpesviruses continue to impact global health across oncology, autoimmunity, and zoonotic risk, this conserved gB epitope may prove to be the key to achieving durable disease control.

## AUTHOR CONTRIBUTIONS

Cong Sun conceived the manuscript. Zi‐Ying Jiang and Peng‐Lin Li contributed equally to the manuscript draft and figure generation. Chu Xie provided revision suggestions.

## CONFLICT OF INTEREST STATEMENT

The authors declare no conflict of interest.
